# Association Between In-Hospital Applications for Long-Term Care Services and Hospital Length of Stay Among Older Adults: Ecological Cross-Sectional Study

**DOI:** 10.2196/76782

**Published:** 2025-09-08

**Authors:** Naoki Takashi, Joji Onishi, Misaki Fujisawa, Tomoko Ohura, Shosuke Ohtera

**Affiliations:** 1 Department of Health Economics Center for Gerontology and Social Science National Center for Geriatrics and Gerontology, Research Institute Obu Japan; 2 Evidence-based Long-term Care Team Center for Gerontology and Social Science National Center for Geriatrics and Gerontology, Research Institute Obu Japan

**Keywords:** length of hospital stay, long-term care insurance, older inpatients, ecological study, cross-sectional study, transitional care

## Abstract

**Background:**

Delayed discharge among older patients presents a major challenge for the efficiency of health service delivery. Prolonged hospitalizations limit bed turnover, increase costs, and reduce the availability of hospital resources. In Japan, older adults must undergo a formal care needs certification process to access public long-term care (LTC) services. Initiating this process during hospitalization is considered ideal for ensuring continuity of care. However, the relationship between the timing of LTC certification applications and hospital length of stay (LOS) remains unclear.

**Objective:**

This study examined the association between the timing of LTC certification applications—specifically those submitted during hospitalization—and average LOS among older inpatients across Japanese prefectures.

**Methods:**

We conducted an ecological cross-sectional analysis using data from all 47 prefectures in Japan for fiscal year 2020. The exposure variable was the proportion of LTC certification applications submitted during hospitalization among all new LTC applications in each prefecture. Exposure data were sourced from the Long-Term Care Database Open Data (Kaigo DB Open Data). The outcome was average LOS among individuals aged ≥65 years at the prefectural level from the 2020 Patient Survey. Linear regression models were used to evaluate the association between the exposure and outcome variables adjusting for relevant covariates. Prefecture-level covariates included proportion of residents living alone, with cognitive decline, or with higher dependency; the proportion requiring dialysis or a respirator before application; the number of health care providers per 100 beds; and the number of nursing and care home beds per 1000 LTC recipients. Sensitivity analyses were conducted using alternative LOS data sources (eg, 2018 and 2020 Hospital Report and 2017 Patient Survey).

**Results:**

The median proportion of in-hospital LTC certification applications was 30.5% (IQR 24.5%-36.1%). The median LOS for older adults was 40 (IQR 37-45.5; range 30-82) days. Prefectures with a higher proportion of in-hospital applications had substantially longer average LOSs. In univariate analysis, the association was statistically significant (β=0.04; *P*=.003), indicating that a 1% increase in in-hospital applications was associated with an approximately 2-day increase in average LOS. This association remained statistically significant after adjustment for all covariates in multivariate models (β=0.06; *P*=.04). Findings were consistent across sensitivity analyses.

**Conclusions:**

Although initiating LTC certification during hospitalization is essential for supporting timely discharge, our findings indicate a positive association with extended hospital stays. This may reflect systemic delays in the certification process. Even with ideal discharge planning, such delays could extend hospitalization and lead to suboptimal allocation of health care resources. As this study was ecological in design, the findings should be interpreted cautiously. Further individual-level data research is warranted to clarify the mechanisms and inform strategies for improving transitional care efficiency in aging populations.

## Introduction

### Background

Hospital length of stay (LOS) is an essential indicator of the efficiency of health service delivery. Optimal discharge timing can enhance patient flow, freeing up beds and health care staff time [1]. Shortening LOS is often associated with improved patient outcomes. A previous study in the United States found that reduced hospital LOS did not increase readmissions or mortality, demonstrating that efficiency improvements can maintain or enhance patient care quality [2].

Delayed discharge, sometimes called *bed blocking*, has been a serious problem in health service delivery for the past half-century [3]. Older patients are particularly at risk of delayed hospital discharge because of their complex health and social needs [4]. When a patient stays in a hospital bed longer than necessary, limited health care resources are consumed, leading to the cancellation of elective surgeries, extending waiting lists, and hindering the admission of emergency cases [5]. Delayed discharge incurs additional costs. A previous systematic review estimated the mean cost per delayed discharge at US $7020, including only hospitalization costs, after adjusting for purchasing power parity [6].

Developing a discharge plan and coordinating the care services that patients will use after discharge from the early phase of hospitalization is essential for facilitating timely discharge. Previous studies have identified causes of delayed discharge such as complexity in arranging care services after discharge, waiting for admission to institutions such as community hospitals, and challenges in nursing home care and long-term care (LTC) facilities [4,7-9]. Adequate discharge planning, including organizing discharge and postdischarge services, reduces LOS [10].

The Japanese government launched the long-term care insurance (LTCI) system in 2000 [11]. As part of Japan’s universal health coverage framework, the LTCI system allows all individuals aged ≥65 years to access publicly funded LTC services, including home-based care (eg, home visit nursing and care), community-based services (eg, adult day services), and institutional care (eg, nursing homes and LTC facilities). To use these services, older adults must undergo a formal care needs certification process administered by municipal governments. This assessment evaluates physical and cognitive function to determine eligibility and assign an appropriate level of care [11,12].

### Objectives

As noted previously, it is important for effective transitional care that discharge planning and coordination of postdischarge services begin during hospitalization. In this context, initiating the care needs certification process while the patient is still in the hospital is considered ideal as it may facilitate a smoother transition to LTC services. However, it remains unclear whether the timing of LTC certification applications—particularly those submitted during hospitalization—is associated with LOS. To generate hypotheses about this potential relationship, this study conducted an ecological analysis using prefectural-level data from Japan. These results are expected to provide a foundation for future research using national databases that include individual-level data.

## Methods

### Study Design

This ecological cross-sectional study was conducted at the prefectural level to explore the association between the timing of LTC certification applications—specifically those submitted during hospitalization—and LOS. All data used in this study were aggregated at the level of Japan’s 47 prefectures and obtained from publicly available government databases. These datasets are accessible online, and detailed information, including URLs, is provided in the *Data Availability* section of this paper. The analysis was conducted in June 2024 using the most recent datasets available at that time in which the exposure and outcome data were from the same reference year.

This manuscript adheres to the Strengthening the Reporting of Observational Studies in Epidemiology guidelines for reporting cross-sectional studies.

### Ethical Considerations

This study was determined to be *nonapplicable* to ethics review by the ethics committee of the National Center for Geriatrics and Gerontology (approval 1772) in accordance with Article 6, Section 1, of the Ethical Guidelines for Medical and Biological Research Involving Human Subjects in Japan. This study used only aggregated, publicly available secondary data at the prefectural level; therefore, informed consent was not required. No direct human participation took place, and no individual-level or identifiable data were used.

### Measurements and Data Sources

#### Exposure Variable: LTC Certification Applications Submitted During Hospitalization

We focused on the timing of LTC certification applications, specifically those submitted during hospitalization. For each prefecture, we calculated the proportion of LTC certification applications submitted during hospitalization in fiscal year (FY) 2020. This proportion was calculated for each of Japan’s 47 prefectures using the following formula: proportion of LTC certification applications submitted during hospitalization = (number of applications submitted during hospitalization in FY 2020 in the prefecture)/(total number of new LTC certification applications in FY 2020 in the prefecture).

In this study, *hospitalization* refers to admissions to hospital beds in Japan, excluding LTC beds covered under medical care insurance.

These data were extracted from open LTCI claims data [13]. These open data were aggregated from the LTCI claims database for each FY beginning in 2018 and are open source and publicly accessible. The Japanese LTCI system was established in 2000 with a standardized electronic claims system. The LTCI claims database contains comprehensive national administrative data because of universal health coverage for LTC and the electronic claims system [14]. The data used for FY 2020 were publicly released in November 2023.

#### Outcome Variable: Average LOS Among the Population Aged ≥65 Years

##### Overview

Average LOS was measured using the average LOS among the population aged ≥65 years discharged from general hospitals in each prefecture in FY 2020. Data were obtained from an open government database―the Patient Survey (government statistics code: 00450022). This is a fundamental statistical survey conducted by the Japanese government to obtain basic data for medical administration by clarifying the actual situation of patients who use hospitals and clinics and their attributes, conditions at the time of visit or admission, diagnoses, and estimations of patient numbers by region [15].

##### Patient Survey

The target was all hospitals nationwide; those with ≥500 beds were surveyed, whereas those with 20 to 499 beds were stratified and randomly selected from the secondary medical care area, defined as a medical administrative area under the Medical Care Act in Japan. The following groups were stratified: *special functioning hospitals*, *psychiatric hospitals (hospitals with only psychiatric beds)*, *hospitals with only LTC beds*, *regional medical care support hospitals*, and *other*.

The average LOS in this survey refers to the average LOS of patients discharged from the hospital during the survey period (September 1-30, 2020). In total, 6185 medical facilities participated in the survey, and information on >1 million patients was collected. A total of 333 patients in the 2020 survey had unknown admission and discharge dates.

The Patient Survey is conducted every 3 years, and the results for FY 2020 were publicly released on June 30, 2022.

##### Covariates

###### Overview

Relevant covariates were selected based on clinical experience, data availability, and findings of previous studies. Previous studies have identified factors associated with LOS or delayed discharge, including hospital staffing [16,17]; living status [7,8]; dementia or cognitive impairment [10,18]; functional dependence [19,20]; specific clinical conditions such as requiring a feeding tube, dialysis, or respirator; and greater pressure ulcer risk [21]. A previous study in the United Kingdom suggested that increasing the supply of nursing and care at home was associated with fewer delayed discharges [22].

###### Hospital Staffing

This was measured as the number of health care providers per 100 beds in each prefecture in FY 2020, with data obtained from the Survey of Medical Institutions (government statistics code: 00450021).

###### Living Status

This was the proportion of older adults living alone in the number of households in each prefecture, which was calculated for each prefecture as follows: proportion of older adults living alone = (number of older people living alone in FY 2020 in the prefecture)/(total number of households in FY 2020 in the prefecture).

Data were extracted from the national census (government statistics code: 00200521).

###### Cognitive Impairment

This is the proportion of newly certified LTC recipients with cognitive decline in each prefecture, calculated using the following formula: proportion of newly certified LTC recipients with cognitive decline = (number of newly certified LTC recipients with cognitive decline in FY 2020 in the prefecture)/(total number of people newly certified for LTC needs in FY 2020 in the prefecture).

The number of individuals with cognitive decline was determined using the grade of activities of daily living (ADLs) related to dementia, which comprises 8 levels: 0, I, IIa, IIb, IIIa, IIIb, IV, and M. Cognitive decline was defined as grade I or higher based on a previous study that proposed grade I or IIa as the cutoff for identifying dementia [23]. Data were obtained from the open LTCI claims database.

In Japan, to access LTCI services, individuals must apply for certification and undergo a standardized needs assessment. One of the items assessed is the grade of ADLs related to dementia. The assessment is conducted by trained personnel commissioned by local governments typically at the individual’s home, at a hospital, or in another care setting. In this study, we used aggregated prefecture-level data derived from these assessments conducted on LTCI applicants.

In this study, *LTC recipients* refers to individuals who were officially certified as requiring LTC under Japan’s LTCI system regardless of whether they were actually using LTC services.

###### Functional Dependence

This was measured as the proportion of newly certified LTC recipients with higher levels of dependence in each prefecture and was calculated as follows: proportion of newly certified LTC recipients with higher dependence = (number of newly certified LTC recipients with higher dependence in FY 2020 in the prefecture)/(total number of people newly certified for LTC needs in FY 2020 in the prefecture).

The level of dependence was determined using the care needs level, which ranges from 1 to 5, with higher levels indicating greater dependence [18]. A previous study reported the median Barthel index total score by care needs level as follows: score of 85 for level 1, score of 70 for level 2, score of 60 for level 3, score of 30 for level 4, and score of 20 for level 5 [24]. Generally, a Barthel index total score of ≤60 indicates dependence [25]. In addition, older adults certified at care levels of ≥3 are eligible for admission to welfare facilities providing lifelong care because of their higher dependence regarding ADLs [26]. Therefore, we defined care needs levels 3 to 5 as indicative of higher dependence.

In Japan, care needs levels are determined by municipalities based on a standardized needs assessment conducted by trained personnel commissioned by local governments. These levels reflect an individual’s required level of support for daily living and determine their eligibility for as well as the amount and type of services provided under the public LTCI system.

All data used in this study were aggregated at the prefectural level and obtained from the publicly available LTCI claims database.

###### Specific Clinical Status

This was measured as the proportion of newly certified LTC recipients who required either dialysis or a respirator within 14 days before application and was calculated separately for each prefecture as follows: proportion of newly certified LTC recipients who required dialysis within 14 days before application = (number of newly certified LTC recipients who required daily dialysis within 14 days before application in FY 2020 in the prefecture)/(total number of people newly certified for LTC needs in FY 2020 in the prefecture) and proportion of newly certified LTC recipients who required a respirator within 14 days before application = (number of newly certified LTC recipients who required a respirator within 14 days before application in FY 2020 in the prefecture)/(total number of people newly certified for LTC needs in FY 2020 in the prefecture).

Although data on tube feeding and pressure ulcers were available, they were not used because of significant correlations with the proportion of people with higher dependence among those newly certified for LTC needs (*r*=0.5 and 0.7, respectively). Data were extracted from the open LTCI claims database.

###### Supply of Nursing and Care Home Beds

This was measured as the number of nursing and care home beds per 1000 LTC recipients in each prefecture. The calculation was based on the number of nursing and care home beds and the total number of LTC recipients in each prefecture during FY 2020: number of nursing and care home beds per 1000 LTC recipients = (number of nursing and care home beds in FY 2020 in the prefecture)/(total number of LTC recipients in FY 2020 in the prefecture) × 1000.

Data were obtained from the Survey of Institutions and Establishments for Long-Term Care (government statistics code: 00450042) and the Report Survey on the Situation of the Long-Term Care Insurance Service (government statistics code: 00450351).

### Statistical Analysis

All variables were summarized using descriptive statistics, including the mean and median, SD, IQR, and ratio of minimum to maximum. A linear regression model was used to analyze the data. The dependent variable in the model was the average LOS among the population aged ≥65 years in each prefecture during FY 2020. A logarithmic transformation was applied because of the right-skewed distribution of the average LOS. The independent variables included the proportion of LTC certification applications submitted during hospitalization and other covariates. The Akaike information criterion and Bayesian information criterion were used to assess the goodness of fit for each model.

The outcome variable from the 2020 Patient Survey provides the average LOS among the population aged ≥65 years. However, there are several limitations. First, the data reflect the average LOS of patients discharged from hospitals during a specific period (September 1-30, 2020). Second, hospitalization patterns and LOS in 2020 were likely influenced by the COVID-19 pandemic. To address these limitations, we conducted sensitivity analyses using data from the 2018 and 2020 Hospital Report and the 2017 Patient Survey. Although the Hospital Report does not include the average LOS among specific age groups, it covers every hospital nationwide, with data collected monthly throughout the year. The average LOS of curative care beds is available, excluding psychiatric, infectious disease, tuberculosis, and LTC beds. This study used the average LOS of curative care beds to reduce bias derived from the differences in bed functions and focus on the most general types of beds.

A significance level of 5% was used for all tests (2-tailed). All statistical analyses were conducted using R (version 4.3.1; R Foundation for Statistical Computing).

## Results

### Overview

The number of LTC certification applications submitted during hospitalization, their proportion among all new LTC certification applications, and the average LOS among individuals aged ≥65 years are summarized in Table 1.

**Table 1:** Summary statistics of long-term care (LTC) certification applications submitted during hospitalization and hospital length of stay (LOS) among older adults by prefecture (fiscal year 2020; N=47).

Variable	Median (IQR)	Mean (SD)	Range	Maximum-to-minimum ratio
Number of LTC certification applications submitted during hospitalization	5440 (4065-8945)	8989.4 (8281.4)	1850-38,550	20.8
Proportion of LTC certification applications submitted during hospitalization (%)	30.5 (29.1-31.7)	30.5 (2.5)	24.5-36.1	1.5
LOS among individuals aged ≥65 y (d)	40 (37-45.5)	43 (10.7)	30-82	2.7

### Bivariate Association

A higher proportion of LTC certification applications submitted during hospitalization was significantly associated with longer hospital stay (β=0.04, 95% CI 0.01–0.06; *P*=.003).

### Multivariate Association

The multivariate association between the exposure and outcome variables is shown in Table 2. A higher proportion of LTC certification applications submitted during hospitalization was significantly associated with a longer average LOS among individuals aged ≥65 years after adjusting for covariates.

The results of the sensitivity analyses using data from the Hospital Report (2018 and 2020) and the Patient Survey (2017) are provided in Tables S1-S3 in Multimedia Appendix 1, supporting the findings of the primary analysis.

**Table 2:** Results of the multivariate linear regression analysis of the association between long-term care (LTC) certification applications submitted during hospitalization and average hospital length of stay among older adultsa.

Characteristic		β (95% CI)	*P* value
Proportion of LTC certification applications submitted during hospitalization		0.06 (0.00 to 0.12)	.04
Number of health care providers per 100 beds			
	Nurses	−0.04 (−0.12 to 0.03)	.30
	Rehabilitation therapists	0.04 (−0.03 to 0.10)	.20
Proportion of older adults living alone		0.05 (−0.02 to 0.13)	.20
Proportion of newly certified LTC recipients with cognitive decline		0.05 (−0.03 to 0.12)	.20
Proportion of newly certified LTC recipients with higher dependence		−0.03 (−0.09 to 0.03)	.40
Proportion of newly certified LTC recipients who required dialysis within 14 d before application		0.07 (0.00 to 0.13)	.049
Proportion of newly certified LTC recipients who required a respirator within 14 d before application		0.02 (−0.05 to 0.08)	.60
Number of nursing and care home beds per 1000 LTC recipients		−0.02 (−0.09 to 0.04)	.40

^a^Average length of stay was logarithmically transformed.

## Discussion

### Principal Findings

Our results indicate that a higher proportion of LTC certification applications submitted during hospitalization was associated with a longer average LOS at the prefecture level. The primary analysis used average LOS data from the 2020 Patient Survey as the outcome variable. However, relying solely on this data source may introduce bias due to its data collection methods and period.

To address this potential bias, we conducted sensitivity analyses using average LOS data from multiple sources, including the Hospital Report (2018 and 2020) and the 2017 Patient Survey. These sensitivity analyses supported the primary results and enhanced the robustness of our findings, reinforcing the observed association between the timing of certification applications and average LOS.

### Comparison With Previous Work

Discharge planning and coordination of postdischarge services starting during hospitalization are essential for effective transitional care. A meta-analysis has shown that such planning, including the arrangement of postdischarge services, can reduce LOS [10]. However, contrary to this expectation, our findings showed that prefectures with a higher proportion of in-hospital LTC certification applications had longer average LOS.

### Possible Explanation: Systemic Delays in the LTC Certification Process

One possible explanation for our findings is that this reflects delays in the LTC certification process itself. In recent years, significant delays have been reported in many municipalities [27]. Several news sources have highlighted that these delays are driven by a rapid increase in the number of applications, which has strained administrative capacity. Indeed, Japan’s increasing aging population continues to drive up the demand for LTC certification [28]. The care needs level for each applicant is determined by a municipal committee based on multiple sources, including face-to-face assessments of physical and cognitive function [11,12,29]. These assessments are time and labor intensive, and as the volume of applications increases, so does the workload for investigators, which may further prolong the certification process. Therefore, delays in certification may hinder the timely arrangement of postdischarge LTC services, potentially contributing to extended hospital stays. Previous studies have noted that discharge delays are often related to challenges in arranging postacute care services [9]. Therefore, although initiating the LTC certification process during hospitalization is generally considered a good practice to support a smooth transition to community-based care, this intended benefit may not be realized when systemic delays are present.

### Alternative Explanations and Considerations

Although our model accounted for several relevant covariates, some unmeasured or residual confounding likely remains, and causal inference is limited by the study’s ecological design. There are 3 alternative explanations and considerations.

First, the number of in-hospital certification applications may simply reflect the presence of patients who are expected to have longer hospital stays. For example, those with multiple chronic conditions, functional dependence, or cognitive impairment or those living alone are more likely to need LTC services after discharge [7-9,19-21]. Because of their longer stays, these patients may have more opportunities to initiate the certification process while still hospitalized. Given the ecological nature of our study, reverse causality cannot be ruled out. Therefore, further investigation using individual-level data is essential to clarify the relationship between the timing of LTC certification application and hospital LOS.

Second, the inclusion of rehabilitation wards in the hospital dataset used to calculate average LOS and in-hospital LTC certification application rates may help explain the observed association. In Japan, these facilities typically provide longer inpatient care than acute care hospitals. For example, patients with stroke may stay for up to 150 days, with an average LOS of 67.5 days compared to 29.5 days in acute care hospitals [30]. In such settings, patients are generally in stable medical condition, and the extended LOS may increase opportunities to initiate LTC certification during hospitalization. Thus, prefectures with a higher concentration of rehabilitation wards may tend to have both longer LOSs and a higher proportion of in-hospital LTC certification applications.

Third, prefectures with longer LOSs may also have fewer available institutional care beds. In such areas, LTC certification applications may be submitted during hospitalization out of necessity, but discharge may be delayed due to a lack of available placements. Waiting for admission to a nursing home has been identified as a key factor contributing to prolonged hospital stays in previous studies [7-9,22]. Japan faces serious shortages in institutional LTC capacity. A document in 2022 reported that 275,000 people were on waiting lists for public nursing homes [31]. Therefore, variation in LTC resource availability across prefectures may help explain the association observed in this study.

### Limitations

This study has some limitations. As an ecological study, confounder adjustment may not have been sufficient because of unmeasured confounders, particularly at the individual level. Consequently, the findings cannot be generalized to individual-level associations because of the potential for ecological fallacy. For example, patients with complex care needs may have longer hospital stays and may also be more likely to submit LTC certification applications during hospitalization. Therefore, these results should be interpreted with caution, and further analyses using individual-level data are strongly warranted.

### Policy Implications

To our knowledge, this is the first study to examine the association between LTC certification application during hospitalization and the average length of hospital stay, offering important insights for policymakers aiming to strengthen the continuity of transitional care in aging societies such as Japan. Our findings indicate that initiating LTC certification during hospitalization is associated with longer hospital stays, which may reflect systemic delays that hinder discharge coordination and lead to inefficient use of hospital resources. Therefore, prefectures with particularly high proportions of in-hospital applications may benefit from targeted interventions to accelerate the certification process.

Although we used the most recently available data at the time of analysis (June 2024), there was an inevitable time lag between data collection and public release—for example, the 2020 open LTCI claims data were published in November 2023, and the 2020 Patient Survey was published in June 2022. Importantly, at a June 2025 meeting of the Ministry of Health, Labour, and Welfare’s LTCI subcommittee, hospital representatives reported that delays in the LTC certification process may be contributing to unnecessary extensions of hospital stays [32]. This contemporary policy discussion underscores the continued relevance of our findings despite the temporal gap.

Several digital transformation initiatives are already underway in Japan to address these challenges [33,34]. These include artificial intelligence–assisted assessment tools; digitized application workflows; remote operation of certification review meetings; and the development of shared, interoperable information platforms among hospitals, insurers (municipalities), LTC service providers, and care recipients. Such innovations have the potential not only to improve the efficiency and responsiveness of the LTC system but also to enhance health care delivery by facilitating timelier discharge planning and reducing unnecessary hospital stays.

### Conclusions

Our study suggests a possible association between LTC certification application during hospitalization and hospital LOS at the prefectural level in Japan. These findings may reflect underlying system-level factors related to discharge coordination and administrative processes. Further research using individual-level data is needed to clarify the mechanisms and examine how LTC certification application timing may influence discharge outcomes. Policymakers in aging societies may benefit from considering these findings as they work toward improving the efficiency and continuity of transitional care.

## Appendix

Multimedia Appendix 1 Results of the sensitivity analyses.

## Abbreviations

ADL: activity of daily living
FY: fiscal year
LOS: length of stay
LTC: long-term care
LTCI: long-term care insurance

## Data Availability

The datasets analyzed during this study can be accessed through the following publicly available resources. All data used in this study are open source and publicly accessible through government repositories [13,15,35-39].
