# Supplementary material for: Association Between In-Hospital Applications for Long-Term Care Services and Hospital Length of Stay Among Older Adults: Ecological Cross-Sectional Study
Source: JMIR Form Res. 2025 Sep 8;9:e76782. doi: 10.2196/76782 (PMC12455142; doi:10.2196/76782)
Supplement: Multimedia Appendix 1 [file formative_v9i1e76782_app1.docx]

**Multimedia Appendix 1**

**Table S1. Results of multivariate linear regression analysis for the association between application for certification during hospitalization and average length of hospital stay in the population aged 65 years or older in 2017**

| **Characteristic** | | **Beta** | **95% CI** | **p** |
| --- | --- | --- | --- | --- |
| Proportion of LTC certification applications submitted during hospitalization | | 0.07 | 0.02, 0.12 | 0.011 |
| Number of healthcare providers per 100 beds | Nurses | -0.05 | -0.12, 0.02 | 0.2 |
|  | Rehabilitation therapists | -0.02 | -0.08, 0.03 | 0.4 |
| Proportion of older adults living alone | | 0.08 | 0.01, 0.14 | 0.023 |
| Proportion of newly certified LTC recipients with cognitive decline | | 0.05 | -0.01, 0.12 | 0.10 |
| Proportion of newly certified LTC recipients with higher dependence | | -0.05 | -0.11, 0.00 | 0.063 |
| Proportion of newly certified LTC recipients who required dialysis within 14 days before application | | 0.06 | 0.00, 0.12 | 0.045 |
| Proportion of newly certified LTC recipients who required a respirator within 14 days before application | | -0.01 | -0.06, 0.05 | 0.8 |
| Number of nursing and care home beds per 1,000 LTC recipients | | 0.01 | -0.05, 0.06 | 0.8 |

CI, confidence interval; average length of stay was logarithmically transformed.

**Table S2. Results of multivariate linear regression analysis for the association between application for certification during hospitalization and average length of hospital stay with respect to curative care beds in 2020**

| **Characteristic** | | **Beta** | **95% CI** | **p** |
| --- | --- | --- | --- | --- |
| Proportion of LTC certification applications submitted during hospitalization | | 0.03 | 0.00, 0.05 | 0.024 |
| Number of healthcare providers per 100 beds | Nurses | -0.01 | -0.04, 0.03 | 0.7 |
|  | Rehabilitation therapists | 0.01 | -0.02, 0.04 | 0.5 |
| Proportion of older adults living alone | | 0.02 | -0.01, 0.05 | 0.3 |
| Proportion of newly certified LTC recipients with cognitive decline | | 0.02 | -0.01, 0.05 | 0.15 |
| Proportion of newly certified LTC recipients with higher dependence | | -0.01 | -0.03, 0.02 | 0.5 |
| Proportion of newly certified LTC recipients who required dialysis within 14 days before application | | 0.01 | -0.02, 0.04 | 0.4 |
| Proportion of newly certified LTC recipients who required a respirator within 14 days before application | | -0.02 | -0.05, 0.00 | 0.10 |
| Number of nursing and care home beds per 1,000 LTC recipients | | 0.01 | -0.01, 0.04 | 0.3 |

CI, confidence interval; average length of stay was logarithmically transformed.

**Table S3. Results of multivariate linear regression analysis for the association between application for certification during hospitalization and average length of hospital stay with respect to curative care beds in 2018**

| **Characteristic** | | **Beta** | **95% CI** | **p** |
| --- | --- | --- | --- | --- |
| Proportion of LTC certification applications submitted during hospitalization | | 0.03 | 0.01, 0.06 | 0.010 |
| Number of healthcare providers per 100 beds | Nurses | 0.00 | -0.04, 0.03 | >0.9 |
|  | Rehabilitation therapists | 0.00 | -0.03, 0.03 | >0.9 |
| Proportion of older adults living alone | | 0.02 | -0.01, 0.05 | 0.2 |
| Proportion of newly certified LTC recipients with cognitive decline | | 0.02 | -0.01, 0.05 | 0.2 |
| Proportion of newly certified LTC recipients with higher dependence | | -0.01 | -0.03, 0.02 | 0.6 |
| Proportion of newly certified LTC recipients who required dialysis within 14 days before application | | 0.01 | -0.02, 0.04 | 0.5 |
| Proportion of newly certified LTC recipients who required a respirator within 14 days before application | | -0.02 | -0.05, 0.01 | 0.12 |
| Number of nursing and care home beds per 1,000 LTC recipients | | 0.01 | -0.02, 0.04 | 0.4 |

CI, confidence interval; average length of stay was logarithmically transformed.
